# Safety of Herbal Medicinal Products: Echinacea and Selected Alkylamides Do Not Induce CYP3A4 mRNA Expression

**DOI:** 10.1093/ecam/nep174

**Published:** 2010-09-15

**Authors:** Maryam Modarai, Elisabete Silva, Andy Suter, Michael Heinrich, Andreas Kortenkamp

**Affiliations:** ^1^Centre for Pharmacognosy and Phytotherapy, The School of Pharmacy, University of London, 29/39 Brunswick Square, London, WC1N 1AX, UK; ^2^Centre for Toxicology, The School of Pharmacy, University of London, 29/39 Brunswick Square, London, WC1N 1AX, UK; ^3^Bioforce AG, CH-9325 Roggwil, Switzerland

## Abstract

A major safety concern with the use of herbal medicinal products (HMP) is their interactions with conventional medicines, which are often mediated via the cytochrome P450 (CYP) system. Echinacea is a widely used over-the-counter HMP, with proven immunomodulatory properties. Its increasing use makes research into its safety an urgent concern. Previously, we showed that Echinacea extracts and its alkylamides (thought to be important for Echinacea's immunomodulatory activity) mildly inhibit the enzymatic activity of the main drug metabolising CYP isoforms, but to this date, there is insufficient work on its ability to alter CYP expression levels. We now report for the first time the effect of a commercial Echinacea extract (Echinaforce) and four Echinacea alkylamides on the transcription of the major drug metabolizing enzyme CYP3A4. HepG2 cells were exposed for 96 h to clinically relevant concentrations of Echinaforce (22, 11.6 and 1.16 **μ**g mL^−1^) or the alkylamides (1.62 and 44 nM). CYP3A4 mRNA levels were quantified using real-time reverse transcription polymerase chain reaction (RT-PCR). Neither Echinaforce nor the alkylamides produced any significant changes in the steady-state CYP3A4 mRNA levels, under these conditions. In contrast, treatment with 50 **μ**M rifampicin resulted in a 3.8-fold up-regulation over the vehicle control. We conclude that Echinaforce is unlikely to affect CYP3A4 transcriptional levels, even at concentrations which can inhibit the enzymatic activity of CYP3A4. Overall, our data provides further evidence for the lack of interactions between Echinacea and conventional drugs.

## 1. Introduction

Interactions between herbal medicinal products (HMP) and conventional drugs are a major safety concern. One of the main pathways for such interactions is via the cytochrome P450 (CYP) enzymes. Direct inhibition or induction of CYP isoforms by HMP can alter the metabolism of conventional drugs, leading to adverse effects. An example showcasing the significance of such interactions is St. John's Wort, which has been shown to affect the pharmacokinetics of several important drugs [1], highlighting the need for *in vitro* investigations into the possible interactions of HMP with CYP isoforms [2].

Echinacea is a popular HMP, well known for its immunomodulatory properties and used worldwide for the treatment of upper respiratory tract infections [3–5]. Previously, we investigated in detail the ability of Echinacea to inhibit various CYP isoforms [6]. We showed that Echinacea and some of its alkylamides weakly inhibit a number of major CYP isoforms. We also screened 10 commercially available Echinacea liquid preparations (ELP) for CYP3A4 inhibitory activity and found that it varied considerably (IC_50_ values 12.7–1817 *μ*g mL^−1^) [6].

Aside from direct enzymatic inhibition, HMPs can affect CYP activity by altering their transcriptional activity (i.e., induce or suppress CYP expression). Altering CYP expression will affect drug metabolism in three different ways. It can alter drug elimination, pro-drug activation, or drug bioactivation (i.e., conversion to toxic metabolites), all of which can have serious consequences [7, 8].

**Figure 1:**
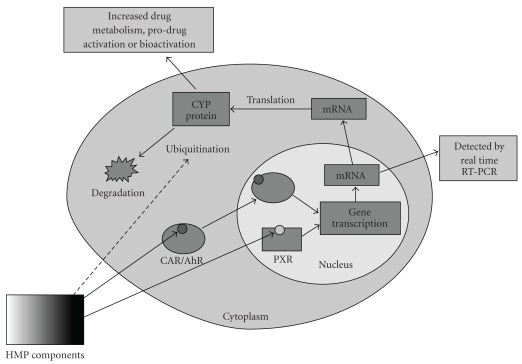
The control of CYP protein levels by HMP. Components of the HMP can either bind the AhR and CAR causing their translocation to the nucleus, or bind the inactive PXR in the nucleus and activate it. Following this binding step, AhR/CAR and PXR can initiate a sequence of events that increases CYP mRNA production and consequently increased CYP protein production. Increased CYP protein levels can accelerate drug metabolism, pro-drug activation and bioactivation. CYP3A4 transcription is mainly controlled by PXR. Increase in mRNA levels can be detected by quantification with real time RT-PCR. Another possibility is that HMP components may also affect post translational modifications such as ubiquitination, which results in protein degradation. This can lead to a change in CYP protein levels without affecting mRNA levels.

CYP gene induction may occur via the action of three intracellular receptors: the aryl hydrocarbon receptor (AhR), the constitutive androstane receptor (CAR) and the pregnane X receptor (PXR) [7, 8]. PXR is believed to be the main mediator of CYP3A gene induction [7]. The mechanism of CYP induction is shown in Figure 1. An important example of CYP induction by HMP is St John's Wort (*Hypericum perforatum*), which has been shown to induce CYP3A4 through PXR activation and can significantly decrease the plasma concentration of concurrently used medicines (e.g., ciclosporin indinavir and the oral contraceptive) [9]. Other natural products, which can induce CYP expression, include kava kava (*Piper methysticum* G. Forster), used to treat anxiety and Qing hao (*Artemisia ammua*) used traditionally as an antipyretic [9].

To date, there is limited data on the induction of CYP enzymes by ELP. Hellum and colleagues [10] found that a commercial ELP (Echinagard-Madaus AG) moderately suppressed CYP3A4 expression in primary human hepatocytes. In addition, Gorski et al. [11] found that Echinacea could cause induction of CYP3A4 in intestinal cells, but suppressed hepatic CYP3A4 expression. Both authors directly assayed CYP activity alone, not the protein or mRNA steady-state levels and their results may be influenced by the presence of various Echinacea constituents that can inhibit CYP activity. Thus, the ability of Echinacea to induce CYP expression is still not well described and a more detailed characterization is needed.

**Figure 2:**
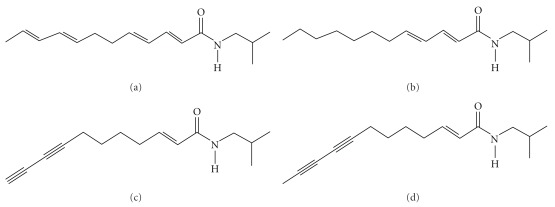
Structures of alkylamides assessed for CYP3A4 mRNA expression: (a) dodeca-2*E*, 4*E*,8*Z*, 10*E*/*Z*-tetraenoic acid isobutylamides (**1**), (b) dodeca-2*E*,4*E*-dienoic acid isobutylamide (**2**), (c) undeca-2*E/Z*-ene-8,10-diyonic acid isobutylamides (**3**) and (d) dodec-2-ene-8,10-diyonic acid isobutylamide (**4**).

We have carried out such an analysis of CYP3A4 induction in human hepatocellular carcinoma HepG2 cells by an ELP (Echinaforce), using real-time reverse transcription polymerase chain reaction (RT-PCR) to determine steady-state mRNA levels. In addition, since alkylamides are important both for the therapeutic activity of Echinacea [12] and its CYP inhibitory potency [13], we investigated whether the isolated alkylamides: dodeca-2*E*,4*E*,8*Z*,10*E/Z*-tetraenoic acid isobutylamide (i), dodeca-2*E*,4*E*-dienoic acid isobutylamide (ii), undeca-2*E/Z*-ene-8,10-diyonic acid isobutylamides (iii) and dodec-2-ene-8,10-diyonic acid isobutylamide (iv) (Figure 2) can induce CYP3A4 transcription in the same system.

## 2. Methods

### 2.1. Chemicals

Hanks' balanced salt solution (HBSS), foetal bovine serum (FBS), minimal essential medium alpha with Glutamax-1 (MEM-*α*) and hexamer random primers were all obtained from Invitrogen (Paisley, UK) Rifampicin HPLC grade, trypsin-EDTA, ethanol, thiazoyl blue tetrazolium bromide (MTT) cell culture tested, RNAse-DNAse free water, dimethylformamide, hydrochloric acid, glacial acetic acid and sodium dodecyl sulphate were purchased from Sigma-Aldrich Ltd (Poole, Dorset, UK). Moloney murine leukemia virus (M-MLV) reverse transcriptase and reverse transcriptase buffer, deoxynucleotide triphosphates (dNTP) and recombinant RNAsin ribonuclease inhibitor were obtained from Promega (Southampton, UK) iQ SYBR Green Supermix was purchased from BioRad Laboratories Inc. (Hertfordshire, UK) and Nucleopsin RNA II kit from Macherey-Nagel (Abgene, Epson, UK). Primers for *β*-actin, glyceraldehyde 3-phosphate dehydrogenase (GAPDH) and CYP3A4 were purchased as high quality, purified OliGold Primers from Eurogentec Ltd. (Hampshire, UK) (Table 1). The alkylamides dodeca-2*E*,4*E*,8*Z*,10*E/Z*-tetraenoic acid isobutylamide (**1**), dodeca-2*E*,4*E*-dienoic acid isobutylamide (**2**), undeca-2*E/Z*-ene-8,10-diyonic acid isobutylamides (**3**) and dodec-2-ene-8,10-diyonic acid isobutylamide (**4**) were purchased from Phytolab GmbH and Co. KG, (Vestenbergsgreuth, Germany). Echinaforce (batch no: 018451) was a kind gift from Bioforce, Switzerland.

### 2.2. Cell Culture

HepG2 cells were a generous gift from Professor Ruth Duncan (Cardiff University, UK). HepG2 cells were routinely cultured in MEM-*α* supplemented with Glutamax and 10% FBS, subcultured at *∼*70% confluence and discarded after 10 passages. The cells were kept in a humidified 37°C incubator with 5% CO_2_.

### 2.3. Cell Viability Assay

The thiazoyl blue tetrazolium bromide (MTT) assay was used to determine cell viability. The assay was performed based on the protocol described by Hansen et al. [14]. HepG2 cells were seeded at a density of 5000 cells per well in flat bottomed 96-well microtitre plates (NUNC, Fisher Scientific, Leicestershire, UK) in 100 *μ*l of medium and allowed to attach for 48 h. The medium was then removed and replaced with fresh medium containing the test substance. The ethanol carried over from the extract or the alkylamide stock solutions was kept below 0.1% to prevent enzyme denaturation and cytotoxicity. 1 : 3 serial dilution series was made from working stock solutions of the Echinacea extract (Echinaforce) and the alkyalmides. For Echinaforce two different working solutions were prepared: 11.6 and 64 *μ*g mL^−1^. The 11.6 *μ*g mL^−1^ working solution was prepared by simply diluting neat Echinaforce with media. To prepare the 64 *μ*g mL^−1^ working solution, while keeping the ethanol content below 0.1%, Echinaforce was dried in a rotary evaporator, freeze dried, re-dissolved in ethanol to yield a concentration of 85.85 mg mL^−1^ and then diluted to 64 *μ*g mL^−1^ with media. Alkylamide working solutions were prepared by diluting ethanolic stock solutions to 3 *μ*M with media, keeping ethanol concentration at 0.1%. A solvent control containing just medium with 0.1% ethanol was also included. Fresh test substance-containing medium was added daily for 4 days. Then the medium was removed, prior to adding 100 *μ*l assay media and 20 *μ*l MTT solution (5 mg mL^−1^ in phosphate buffered saline). The plate was incubated at 37°C for 4 h before adding 150 *μ*l of solubilization solution (20% SDS 40% DMF, 2% glacial acetic acid and 1% vol/vol HCl in distilled water, pH 4.7) and leaving the plate to develop overnight. Formazan production was quantified by determining the absorbance at a wavelength of 560 nm using a plate reader (Labsystems Multiscan, VWR International).

### 2.4. Cell Treatment for Induction Studies

300 000 cells were seeded in 25 cm^2^ tissue culture flasks and left to adhere for 24 h. The medium was then replaced with fresh medium containing the test-substance (Echinaforce: 22, 11.6, 1.16 *μ*g mL^−1^, alkylamides: 1.62, 44 nM). Rifampicin at a concentration of 50 *μ*M was used as a positive control for CYP3A4 activation and the solvent control contained medium with 0.1% ethanol. The test substance containing medium was prepared by diluting neat Echinaforce, a stock solution of alkylamide, or a stock solution of rifampicin directly into the media ensuring that the concentration of ethanol did not exceed 0.1%. Fresh test substance-containing medium was added daily for 4 days, before RNA isolation for real-time RT-PCR.

### 2.5. RNA Isolation

Cells were harvested using trypsin–EDTA and total RNA was extracted using the Nucleospin RNA II kit, according to the manufacturer's instructions. The RNA was quantified spectrophotometrically by measuring absorbance (Abs) at 260 nm (NanoDrop ND 1000 UV Spectrophotometer). The purity of the final preparations was determined by calculating the ratios Abs 260/280.

### 2.6. Reverse Transcription

For each sample, 2.5 *μ*g of total RNA was reverse transcribed using M-MLV Reverse Transcriptase according to the manufacturer's instructions. Briefly, RNA was diluted to 125 ng mL^−1^, then 7 *μ*l of 5× reaction buffer, 4 *μ*l dNTP (10 mM each), 1 *μ*l RNase inhibitor and 1 *μ*l hexamer primers were added to 20 *μ*l of diluted RNA on ice. The mixture was heated at 65°C for 10 min and then snap-cooled on ice for 2 min, prior to the addition of 2 *μ*l of reverse transcriptase (200 units *μ*l^−1^). The reaction was carried out at 42°C for 90 min. The cDNA was stored at −80°C until further use.

### 2.7. Real-Time PCR

The sequences of the primers used for real-time PCR are shown in Table 1 along with the Genebank accession numbers of the cDNA sequences used for primer design. Primer selection was accomplished by using the Beacon designer 5.1 software suite (Premier Biosoft International, Palo Alto USA). The primer concentration was optimized to achieve 98–100% amplification efficiency (data not shown).

Real-time PCR analysis was carried out using an iCycler iQ optical system multicolour real-time PCR detection system with the iCycler optical system software version 3.1 (Bio-rad Laboratories Inc). The samples were prepared by mixing 10 *μ*M of each primer, 25 *μ*l 2× iQ SYBR Green Supermix, 2 *μ*l of 1 : 10 diluted cDNA and the appropriate volume of nuclease-free water. Two 20 *μ*l aliquots were used for the amplification reaction. All reactions were run in duplicate. The hot start polymerase was activated by heating at 95°C for 3 min. The cycling conditions were: 0.1 min at 95°C (melting) and 0.45 min at 55°C (annealing and extension). Threshold values (*C_t_*) were calculated automatically by the software.

The *C_t_* data was processed according to the method described by Pfaffl [15]. Briefly, the Pfaffl equation [15] was first used to calculate the relative gene expression ratio, that is, the change in target gene expression divided by the change in the reference gene expression.

(1) $$R=\frac{E_{\text{target}}^{∆{C_{t_{\text{target}}}}_{}({\text{Mean}}_{\text{control}}-\text{Mea}{\text{n}}_{\text{treatment}})}}{E_{\text{reference}}^{∆{C_{t_{\text{reference}}}}_{}({\text{Mean}}_{\text{control}}-\text{Mea}{\text{n}}_{\text{treatment}})}},$$

where *R* is the relative gene expression ratio, *E* is the amplification efficiency (*E* = 2 at 100% efficiency) and Δ*C_t_* is the difference in threshold (*C_t_*) value.

Then the *R* values were normalized with respect to the vehicle control. Statistical significance was determined using the relative expression software tool (REST), a randomization test developed by Pfaffl et al. [16].

## 3. Results

### 3.1. Determination of Clinically Relevant Concentration Ranges

Based on the results and predictions by other groups [10, 12, 17], we determined that the clinically relevant Echinaforce concentration range lies between 1 and 25 *μ*g mL^−1^. For the alkylamides the clinically relevant concentrations were taken from Woelkart et al. [18, 19], who found that alkylamide (**1**) reached the highest plasma concentration of 44 or 1.62  nM in human volunteers dosed with an *E. angustifolia* root extract and Echinaforce, respectively [18, 19].

### 3.2. Cell Viability Assay

The viability of HepG2 cells exposed to Echinaforce (up to 64 *μ*g mL^−1^), ethanol (up to 0.1%) or each of the alkylamides (up to 3 *μ*M), for 96 h was investigated in the MTT assay. No loss of cell viability was observed.

### 3.3. Real-Time PCR Analysis

**Figure 3:**
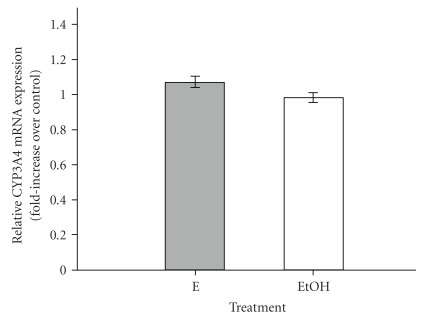
Relative CYP3A4 mRNA expression (fold increase over vehicle control) for *β*-actin using GAPDH as the reference gene, following treatment of HepG2 cells with Echinaforce (E: 22 *μ*g mL^−1^) or with vehicle control [0.1% ethanol (EtOH)] for 96 h. A value of 1 denotes no increase over the control. The values are the average of three independent experiments ± SE.

**Figure 4:**
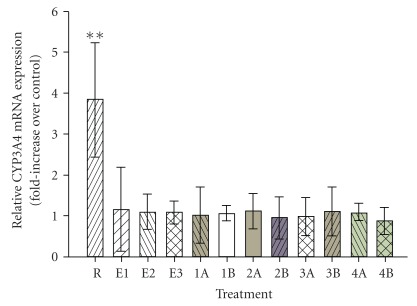
Relative CYP3A4 mRNA expression (fold increase over vehicle control) for CYP3A4 in HepG2 cells after a 96 h treatments with: 50 *μ*M rifampicin (R), Echinaforce (E1: 1.16 *μ*g mL^−1^, E2: 11.6 *μ*g mL^−1^, E3: 22 *μ*g mL^−1^), alkylamide **1** (1A: 1.62 nM, 1B: 40 nM), alkylamide **2** (2A: 1.62 nM, 2B: 40 nM), alkylamide **3** (3A: 1.62 nM, 3B: 44 nM) and alkylamide **4** (4A: 1.62 nM, 4B: 44 nM). A value of 1 denotes no increase over the control. Values are the average of three independent experiments ± SE. Statistically significant results (*P* < .05 with a randomization assay) are marked with **.

To determine if Echinaforce and its alkylamides can change the steady-state mRNA levels of CYP3A4, we exposed HepG2 cells to Echinaforce or alkylamides over a period of 96 h. Rifampicin was used as the positive control and plain medium with 0.1% ethanol was used as the vehicle control. Following treatment, the mRNA was extracted and the levels of CYP3A4 mRNA were quantified by real time RT-PCR using *β*-actin as the internal control (reference gene). It has been reported that Echinaforce can up-regulate *β*-actin in human monocytes/macrophages [12]. Therefore, a second reference gene, GAPDH, was used to detect *β*-actin up-regulation. We found that exposure to Echinaforce (Figure 3) or the alkylamides (data not shown) did not result in up-regulation of *β*-actin expression in HepG2 cells, allowing *β*-actin to be used reliably as an internal control. Melt-curve analysis confirmed the absence of non-specific amplification products (data now shown). The results of the real time PCR analysis are shown in Figure 4 and Table 2. Exposure to rifampicin resulted in a statistically significant (*P* < .05) up-regulation of CYP3A4 expression (3.8-fold) compared to the vehicle control (0.1% ethanol) as shown in Table 2. In contrast, exposure to Echinaforce or the alkylamides did not produce any statistically significant changes (at the 5% level) in the CYP3A4 mRNA levels (see Table 2).

## 4. Discussion

This is the first study to investigate the influence of an Echinacea preparation (Echinaforce) and four alkylamides on the steady state CYP3A4 mRNA level in HepG2 cells. No statistically significant changes in the mRNA steady-state level of CYP3A4 were observed after treating the cells with clinically relevant concentrations of Echinaforce or any of the alkylamides. In contrast, treatment with rifampicin resulted in a 3.8-fold up-regulation of CYP3A4. This value is in agreement with other studies with rifampicin [7, 20]. Therefore, we conclude that the compounds found in Echinaforce and the alkylamides tested have no effect on CYP3A4 transcriptional activity in HepG2 cells. It is possible that Echinaforce or the alkylamides might induce CYP3A4 expression at higher concentrations than the ones we tested. However, this would be at levels well above the clinically relevant range and, thus, would have no practical significance.

The effect of Echinaforce on the induction of other CYP enzymes has not been investigated and the apparent inability of Echinaforce to induce CYP3A4 does not imply that this will be the case for the remaining isoforms. However, due to the prominent role of CYP3A4 in drug metabolism any alteration of CYP3A4 activity is a major contributor to HMP-drug interactions.

It is important to note that we investigated mRNA steady-state levels instead of the actual CYP3A4 protein expression. Therefore, it is not possible to rule out that Echinaforce might affect the post-translational regulation of CYP3A4 expression (Figure 1). Several types of post-translational regulation for CYPs have been documented including ubiquitination, nitration and phosphorylation [21]; however, we are not aware of published work suggesting that CYP post-translational regulation can be affected by HMPs.

Hellum et al. [10] found that two other Echinacea preparations (Echinagard and Madaus AG) caused a small, but significant suppression of CYP3A4 activity in cultured primary human hepatocytes. This discrepancy could be the result of the differences in methodology. Hellum et al. [10] exposed the cells to much higher (4–80 times, i.g., 4.735–473.5 *μ*g mL^−1^) Echinacea *purpurea* concentrations, and assayed enzyme activity instead of determining mRNA levels. We have previously shown [6] that Echinacea preparations can directly inhibit CYP3A4. Hence, it is possible that the reduction in CYP3A4 activity observed [10] is not in fact related to reduced CYP3A4 expression, but rather a direct inhibitory effect on its enzyme activity. A time course study would have to be carried out to distinguish between reduced expression and direct enzyme inhibition.

Although without effect on CYP3A4 mRNA levels, the Echinacea extract (Echinaforce) concentrations tested here were sufficient to cause observable inhibition of CYP3A4 activity. The IC_50_ for CYP3A4 inhibition estimated for Echinacea [6] was 27.2 *μ*g mL^−1^, close to the 22 *μ*g mL^−1^ employed in the present CYP induction study.

HepG2 cells are a human hepatocarcinoma cell line and thus, they do not exactly mirror the CYP profile present in the human liver. However, the mechanism controlling CYP3A4 expression in HepG2 cells are identical to those of human hepatocytes and thus, if Echinaforce does not induce CYP3A4 in HepG2 cells it is highly likely that it will not do so in human hepatocytes either. Therefore, overall, our results suggest that CYP3A4 induction can presumably be excluded as an avenue for interactions between Echinaforce or its alkylamides (the major active constituents) and conventional medicines thus reducing the risk of adverse effects arising from such interactions. Overall, this data improves our understanding of the safety profile of Echinacea. It also shows that Echinacea extracts are unlikely to cause cytochrome P450 mediated interactions with conventional medicines [22]. This is in agreement with the phamacovigilance data, as there is a lack of reports documenting interactions between Echinacea and concurrently used medication.

## Funding

Bioforce, Switzerland and the Maplethorpe Trust (University of London).

## Figures and Tables

**Table 1:** *β*-actin, GAPDH and CYP3A4 primers.

Gene	Gene accession no.	Primer	Sequence	Concentration (nM)	Product size
*β*-Actin	X00351	Forward	5′-TCAGCAAGCAGGAGTATG-3′	300	97
		Reverse	5′-GTCAAGAAAGGGTGTAACG-3′	300	
GAPDH	NM_002046	Forward	5′-TCTCTGCTCCTCCTGTTC-3′	900	120
		Reverse	5′-GCCCAATACGACCAAATCC-3′	900	
CYP3A4	NM_017460	Forward	5′-ATCATTGCTGTCTCCAACCTTCAC-3′	200	103
		Reverse	5′-TGCTTCCCGCCTCAGATTTCTC-3′	200	

Genebank accession numbers, primer sequences, concentrations and product sizes are shown all genes.

**Table 2:** Induction of CYP3A4 gene expression in HepG2 cells by Echinaforce and its alkylamides.

Treatment	Concentration	Relative CYP3A4 mRNA expression^a^	(±) SE
Rifampicin	50 *μ*M	3.83	1.39
Echinaforce	1.16 *μ*g mL^−1^	1.16	1.03
	11.6 *μ*g mL^−1^	1.09	0.44
	22 *μ*g mL^−1^	1.08	0.28
Alkylamide **1**	1.62 nM	1.03	0.69
	44 nM	1.07	0.19
Alkylamide **2**	1.62 nM	1.11	0.44
	44 nM	0.95	0.52
Alkylamide **3**	1.62 nM	0.98	0.46
	44 nM	1.11	0.60
Alkylamide **4**	1.62 nM	1.08	0.22
	44 nM	0.87	0.34

The cells were treated for 96 h, changing the test substance containing medium every 24 h. Values are the average of three independent experiments ± SE.

^a^Fold increase over the vehicle control.
